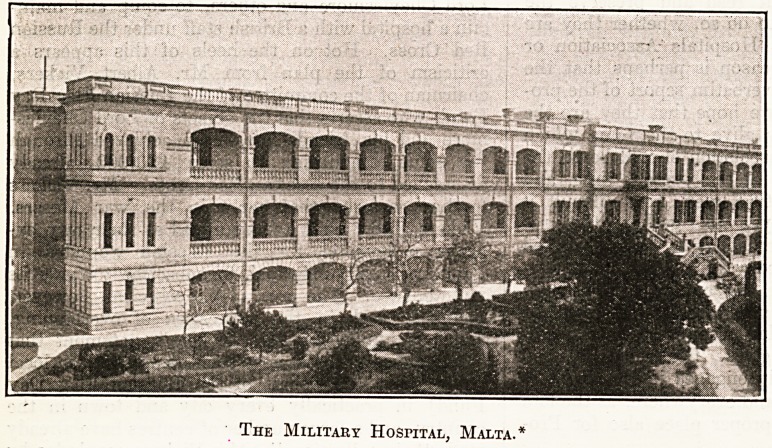# Hospital and Institutional News

**Published:** 1915-08-21

**Authors:** 


### HOSPITAL AND INSTITUTIONAL NEWS.

### OUR NAVAL MEDICAL HEROES.

In the historic Dispatch from Vice-Admiral John M. de Robeck, describing the heroism of all ranks in both Services in connection with the landings on the Gallipoli Peninsula, attention has naturally centred on those who have received the six Victoria Crosses which have been awarded. Two, as we all remember with pride, have been given to midshipmen, or, as the Service affectionately calls, or used to call them, "snotties." In the Dispatch itself Vice-Admiral de Robeck mentions in his list of those officers and men who have performed specially meritorious service, the names of the following medical officer and member of the sick-berth staff:—Surgeon P. B. Kelly, R.N., attached to R.N.A.S., "was," says the official statement, "wounded in the foot on the morning of the 25th in *River Clyde*. He remained in *River Clyde* until morning of 27th, during which time he attended 750 wounded men, although in great pain and unable to walk during the last twenty-four hours." Mention is also made of Tempy. Surgeon W. D. Galloway, of H.M.S. *Cornwallis*, who is among those "commended for service in action."

### SURGEON LANGFORD'S D.S.O.

When we turn to the list of actual awards for bravery we see that among those to whom has been awarded the Companionship of the Distinguished Service Order is Surgeon Martyn Henry Langford, R.N. The official account of his services states that "During the time H.M.S. *Inflexible* was steaming to Tenedos, after having struck a mine, Surgeon Langford brought up the wounded from the fore distributing station in the dark. Fumes permeated the place, rendering five men unconscious. Surgeon Langford, though partially overcome by the fumes, continued his work."

### THE STAR AND GARTER AS A HOME OF REST.

The members of the Auctioneers' and Estate Agents' Institute of the United Kingdom have purchased for £21,500 the Star and Garter Hotel on Richmond Hill, which originally cost £80,000. The difference between cost and purchase price points to a depreciation altogether beyond the natural fall in prices, owing to change of fashion, or in values. The Auctioneers' and Estate Agents' Institute have presented their purchase to the Queen as a permanent home for paralysed and totally disabled sailors and soldiers. Her Majesty, in accepting the gift, has announced her intention to hand over the buildings, terraces, gardens, and grounds to the British Red Cross Society, and to place them under the control and management of that body. This responsibility has been undertaken by the British Red Cross Society, who will equip and maintain at the old Star and Garter an institution devoted to the care of such totally disabled men as are retired from the Services. Despite the proverb, it is sometimes well to look a gift-horse in the mouth, and, having regard to the price paid, and to the notorious fact that the Star and Garter has steadily degenerated for many years in hygienic efficiency and everything which guarantees absolute cleanliness, freedom from animal and insect life, and the comfort and health of residents in and visitors to a great hotel, we feel it essential to inquire whether the Auctioneers' and Estate Agents' Institute of the United Kingdom, as experts in such matters, thoroughly investigated the buildings and their hygienic and sanitary condition, and put the whole of them in perfect order in these respects before they tendered their gift to H.M. the Queen. On the last occasion we were present at a public banquet at the Star and Garter, not so many years ago, we were served, as one of the guests at the high table, with a fine cockroach in our portion of the turtle soup.

### A WHITE ELEPHANT OR A GIFT?

We understood that the above cockroach incident was not an infrequent occurrence, and it points to the presence of insect life in the kitchens and other parts of the buildings in considerable quantities. It further indicates an absence of efficient administration of the structural and sanitary condition of the buildings of the Star and Garter Hotel in the past, the effects of which must be absolutely eradicated before its buildings can be properly used for the reception of totally disabled warriors whom the nation desires to honour in a National Home of Rest. We hope the chairman of the Auctioneers' and Estate Agents' Institute of the United Kingdom will be able to clear up these points satisfactorily, because the work on another great hotel building in Belgravia, rendered necessary from similar causes, entailed an expenditure of many thousands of pounds to make the kitchens and basement free from the cockroach, insect, and rat curse, and to render the chambers and apartments relegated to the guests exempt from such pests, and hygienically pure and habitable.

### THE WANDSWORTH UNION'S INSTITUTIONS AND THE PROGRESS MADE.

Following up our Note on this Union last week, voluntary hospital managers do not yet perhaps realise that the Wandsworth Union from its large area provides a congeries of institutions in which the process of classification, so necessary to the cause of Poor-Law reform, has begun. St. James's Infirmary, under Dr. Maccormac, the house and lying-in block at Swaffield Road, and the home recently taken over by the War Office form a remarkable group in this connection.

### REDUCTIONS IN AN INFIRMARY DIETARY.

Dr. Baly, the medical superintendent of the Lambeth Infirmary, at the outbreak of the war introduced, with the sanction of the board, certain modifications in the dietary of the officers. These consisted chiefly in a reduction of the rations allowed, and an increase of 1s. in the amount for fruit and vegetables, at the same time giving the matron greater freedom as to what was purchased under this head, and from where it was to be obtained. These changes have proved most successful, as the cost per head per week has for the first time in the history of the infirmary fallen under 10s., the figure being 9s. 10d. Dr. Baly was of opinion that the officers were as well, if not better, fed than they have been in the past, and he, therefore, has extended the principle, increasing the allowance for sundries to 3s. 6d. and further decreasing the rations. The value of the rations eliminated exceeded the extra 2s. 6d., and would give the matron a far greater latitude in feeding the officers. The experiment, so successfully carried out at the Lambeth Infirmary, is worthy of consideration by those Boards of Guardians who, despite the war and its attendant evils in obtaining provisions, have continued the same scales of rations for their officers as were in existence in pre-war days.

### A PANEL DOCTOR ORDERED OUT OF COURT.

The second case within a comparatively short period of panel doctors being charged with failing to attend patients on their panel has occurred in the Battersea district. In the latter instance a married woman had been attended by a Dr. Bernard, a woman doctor connected with a nurses' home at Bridge Road, Battersea, and after she had left, the patient was seized with violent pains in the head. The nurse in charge of the case went to the woman's panel doctor, Dr. E. C. Clanchy, of Battersea Park Road, who, however, did not see his patient until the next day, when she had died. The nurse's story was that the doctor refused to attend, but this he denied, stating that he was not told it was an urgent case. Had he known that, he would have gone the same day. The medical evidence showed that death was due to apoplexy, and that medical attention could not have saved the woman's life. The Coroner, Mr. S. I. Oddie, pointed out to the jury at the inquest that if panel doctors refused to attend their patients they were taking upon their shoulders a great responsibility, and if death had been caused or accelerated by such an act it might lead to the question of manslaughter being raised. It was not, however, raised in this case. The jury, in returning a verdict of "Death from natural causes," added a rider that it was Dr. Clanchy's duty to attend the woman as his panel patient, that he was duly requested to do so, and refused to attend, and they censured him. Dr. Clanchy commenced to protest against this rider, but was stopped by the Coroner, who ordered him out of court.

### MEDICAL TREATMENT FOR SOLDIERS ON FURLOUGH.

In The Hospital for July 24 (p. 355) we referred to the announcement made by Mr. Charles Roberts in the House of Commons recently to the effect that, in collaboration with the War Office, the Insurance Commissioners had arranged for the provision for medical attendance for soldiers on furlough and for soldiers living at home or in billets, and we expressed the hope that such arrangements, which it was stated had already been communicated to the Insurance Committees and Panel Committees, would be made public without delay. We have, up till now, been unable to trace any public announcement on the subject, and upon inquiry at the offices of an Insurance Committee we were astonished to find that no communication had apparently been received in the matter beyond a circular-letter dated February last. In this letter the subject is dealt with only in such a manner as to acquaint the Committee with the general procedure to be adopted for the settlement of the account when a soldier applies to a civilian doctor for treatment. Moreover, the arrangement, to which the letter refers, for utilising Insurance Committees, or Panel Committees, as the channels whereby claims for payment for services rendered should be presented to the War Office appears to be permissive only. These arrangements actually provided should have been made known generally amongst soldiers, and every civilian doctor should also have been informed. Unfortunately, these essential steps have not been taken. It may be well, therefore, to state that the arrangements provide that when a soldier proceeds on furlough he is supplied with a "furlough paper," which instructs him, should he require medical aid, to apply at the nearest military hospital whenever practicable. In the event, however, of the soldier living more than two miles from a military hospital, or his condition being such that he is unable to travel to the hospital, he may, at the cost of the funds of the War Office, call in a civilian doctor. The rates allowed for attendance are stated on the "furlough paper," which the soldier is instructed to show to the doctor. Soldiers who, while training, reside at home or in billets, if they need medical aid, should apply to the medical officer attached to their unit. In emergencies, where such medical officer or a military hospital is not available, the soldier may call in a civilian doctor. The soldier is required to report the matter at once to his commanding officer, while to obtain payment for his services from the War Office the civilian doctor is required to procure and fill in the special Army form O. 1667. This form, when completed on the conclusion of treatment, has to be sent to the Deputy Director of Medical Services for the district. It must, however, be realised that when a soldier applies to a civilian doctor for treatment in circumstances where he should have had recourse to military arrangements, the War Office does not accept responsibility for payment.

### INSURANCE AGAINST AIR RAIDS AT WORCESTER.

At the monthly meeting of the executive committee of the General Infirmary, Worcester, the question of insurance against air raids was discussed. The Chairman moved the adoption of the recommendation of the sub-committee, that the infirmary buildings be insured against damage from air raids in the same sum as against fire—£12,000. In bringing forward the resolution, the Chairman admitted that the damage was slight, but they had to consider that they were responsible for the institution and that they ought to cover every contingency. He understood that with the Nurses' Home and the annexe the amount would be about £20,000. In supporting the resolution, Mr. Bruce Ward remarked that the money would go to State funds, and that was an additional reason why they should not hesitate to insure. The proposal was agreed to. In this connection the committee of Worcester General Infirmary and others who have, for one reason or another, delayed to cover such risk as there may be, will read with interest the article, contributed by an expert, which appears on page 432. Statesmanlike administrators will feel the force of the Chairman's contentions, and a right judgment should readily be formed in the light of our contributor's information.

### THE NEEDS OF THE FRENCH RED CROSS.

Mr. C. A. Lievre, the Consular agent for France at Bradford, in appealing for further funds on behalf of the French Red Cross, has relied not only on local, indeed general, enthusiasm on behalf of our great Western Ally, but also, we understand, on certain facts in France which have been well summarised in the *Yorkshire Observer*. They are worth reproducing for the general benefit. Every Frenchman between the ages of nineteen and forty-eight, we are reminded, has been called to serve his country, and active service claims so many that what is practically a dislocation of ordinary business obtains in the country. Again, the main industrial "productive" portions of France have been over-run, or remain largely in the hands of the enemy, which means a potential loss of vast extent, in spite of the superb reorganisation which has sprung up in other centres to meet the deficiency. Again, the length of line held by the French, and consequently the amount of Red Cross assistance required by the French armies, is, at all events so far as the Western Front is concerned, on a different scale from that required on our own. Lastly, of course, the French field hospitals near the fighting line do not limit their services to Frenchmen, but Belgian and British wounded, where necessary, also can claim their ministrations and services. Sir James Roberts, Bart., has lent his patronage to the appeal, and subscriptions may be sent to Mr. C. A. Lièvre, at North Parade, Bradford.

### ASYLUM AGITATION IN AUSTRALIA.

The Hospital and Asylum Employees' Union is agitating for a Wages Board, writes our Melbourne correspondent. Their argument is that "the healing and nursing of the sick should not be based on unjust conditions." All hospitals and asylums were circularised. At the half-yearly meeting of the Union held at the Trades Hall in the city, it was reported that among many replies only one hospital offered opposition to the appointment of a board. In view of the unrest which obtains among a certain section of mental hospital staffs, to which our correspondence columns this and last week bear witness, the situation in Australia has an interest for students of the situation at home.

### AN R.A.M.C. OFFICER'S DEATH.

Lieut.-Colonel G. A. Edsell, commanding officer of the 1st/3rd Home Counties Field Ambulance, Royal Army Medical Corps (T.F.), died last Sunday night at his house at Surbiton. After being at the Front for several months he fell a victim to pleurisy. Undeterred, however, he continued to remain at his post until it became necessary that he should be invalided home. He received his medical education at "Bart.'s" and University College Hospital. He took the Conjoint Diploma in 1886, and later proceeded to Durham, where he graduated M.D. in 1902. Always keen on his work, in 1905 he obtained the Diploma of Public Health, both of London and Cambridge. Lieut.-Colonel Edsell was an examiner and hon. life member of the St. John Ambulance Association. He had also held the post of Surveyor, Medical Department, at the Admiralty. Among his publications was an article in the *British Medical Journal* on the "Successful Reposition of a Completely Severed Finger."

### SHORTAGE OF DOCTORS IN AUSTRALIA.

So many requests for more doctors have been received within this month by the Minister for Defence, Senator Pearce, from the London War Office that the Universities, writes our Australian correspondent, have been appealed to. Sydney led, by the Senate adopting the Faculty of Medicine's recommendation that the present fifth-year students should be allowed to present themselves for the final degree examination in September 1915, instead of March 1916, and the fourth-year students in March 1916. The alteration will apply only to undergraduates volunteering for active service, but fifty of the fifth-year men and the whole of the fourth-year men (seventy-four) had already volunteered. Adelaide at once fell into line with Sydney, and Melbourne followed later. Sir Harry Allen (Melbourne) has carefully explained that this does not mean any lowering of standard. The general hospitals have been also asked to try to do with as small an indoor staff as possible, and thus release a number of their junior medical officers. Up to June 16 458 medical officers had left Australia for France, Egypt, or Turkey, and of these twenty-eight have been killed; eighty or ninety of these left within the six weeks before the date referred to. Another twenty leave directly, and the drain on the medical profession is likely to be continuous. The latest from the War Office is that it "invites the services of *any number* of suitable, well-qualified Australians, not over forty years of age, in good health, and fitted for the field. Service is also invited from others up to 20 per cent. of the whole, and up to forty-five years of age." The latter are to be appointed to hospital or other military service in the United Kingdom. The following appears to be a warning: "Not more than six men of recognised consultant rank could be appointed to hospitals at home or abroad." Most of our Melbourne specialists or "recognised consultants," as a matter of fact, are now in Egypt, or possibly Turkey. For Australia's own needs at least thirty-six medical officers are at present required for whole-time service (quarantine, etc.), besides those needed for relief and reinforcing medical services in New Guinea and elsewhere. Many more of our medical men would answer to the Empire's call, could they either dispose of their practices or obtain a substitute. The Council of the Victorian British Medical Association have taken up the matter and appointed a committee to deal with it and to facilitate the departure of those of its members who desire to go. This information comes aptly to hand, in view of the situation discussed in our first leading article.

### GERMAN FIRMS WITH ENGLISH PRETENSIONS.

On Monday last Messrs. Knoll and Co., Limited, were fined £5 on each of eleven summonses for infringing the Stamp Duty on Medicines Act, 1812, by selling certain medical preparations without the proper stamp. The case was heard before Sir John Knill at the Mansion House Police Court, and costs amounting to five guineas were imposed, which made a total of £60 5s. In stating the case for the prosecution, counsel remarked that the manager of the company, a certain Walter Braun, was now interned as an alien enemy, and that, therefore, the summonses issued against him should be withdrawn. Although registered as an English company it appeared that the firm was German throughout. Sir John Knill agreed to state a case. Whatever may eventuate, it is not indiscreet to say that some means must be found for preventing German firms from registering as English companies.

### WATER STERILISERS FOR BELGIAN SOLDIERS.

An interesting appeal is being made by the Belgian Soldiers' Fund for money with which to provide sterilisers for the purpose of purifying the water for the Belgian Army. It does not require any scientific imagination—not much imagination of any kind for the matter of that—to realise that as regards water the men now fighting around the canals and rivers of Flanders are more or less in the position of castaways adrift on the sea: "Water, water everywhere, nor any drop to drink." It is mostly, if not all, contaminated. Hence the requirement, made apparently from the Belgian Army Headquarters, for a supply of portable sterilisers. These, and some 400 are asked for, would largely help to supply the needs of each Field Hospital and company. Capable of sterilising from twenty to 100 gallons an hour, and costing, it is understood, £50 each, including what chauffeurs call "spares" and fittings, the pattern decided on is stated to be able to cater for 700 men. Readers of the Special Number of The Hospital on the subject of Transport, which was published on April 17, will recall the descriptions and illustrations there given of field soup kitchens and other mobile units which depended upon such sterilisers for their water supply. The authors of the appeal hope that every town or district will present the cost of one of these sterilisers, which could then be sent to the Front with the words "A gift from . . ." What a contrast in practical value and lively sentiment, too, to that which garnishes tea-cups and saucers with the well-known phrase, "A present from Blackpool," in peace-time! Is not August still the month when such presents as that now appealed for should be bought? The Fund's address is 19 James Street, Oxford Street, W.

### HOSPITAL WAR WORK AT MELBOURNE AND SYDNEY.

The Melbourne Hospital has lately received an additional gift of £10,000 from the "Edward Wilson Estate," writes our Melbourne correspondent, bringing up the total benefaction from that quarter to £145,000. With the exception of a small block not to be built at present, the new hospital will shortly be completed without debt. Increasing demands are being made on space in all the Metropolitan hospitals at present, owing to economic distress consequent on the war, and a suggestion was made by Dr. Smeal, the acting medical superintendent of the Melbourne Hospital, that cases which only required ordinary care and attention could be treated as well in country hospitals, these being seldom full. But at least one country hospital, Maryborough, has offered the Defence Department the use of from fifteen to twenty beds for wounded soldiers. In Sydney the State Government are purchasing a large mansion and grounds, Cranbrook, at Rose Bay, as a home for convalescent soldiers, and Mr. T. A. Dibbs, general manager of the Sydney Commercial Banking Company, has made a free gift of his residence and grounds at North Sydney, estimated market value at least £15,000, as—primarily—a convalescent home for wounded soldiers.

### THEFTS FROM THE LONDON HOSPITALS.

There has been apparently an absence of thefts from the Metropolitan hospitals which speaks well for the additional employees that have been introduced to deal with the sick and wounded soldiers; and it has been asserted that this is the more remarkable when some believe that the great majority of those addicted to petty thefts and larceny have drifted into the ranks of the many services associated with the Army. Hence is explained the reason for the diminution of crime! But there are exceptions to every rule, as the authorities at the London General Hospital discovered. There had been a good many thefts from the hospital lately, and a watch was kept, when a workman was seen leaving with a large bag. An inspection brought to light a pyjama suit, valued at 6s. 6d., which the man told the magistrate, Mr. Lister Drummond, at the South-Western Police Court where he was charged with purloining it, that he had found the pyjamas in a dustbin, lying on a pile of papers which kept them clean. Other mornings he had seen in the dustbin various articles, and thought they were of no use, as they had apparently been thrown away. Mr. Drummond, in fining the man 40s., said that people must get rid of the idea that they could take things which belonged to the Government, even if found in a dustbin. No evidence was given to show how the pyjamas came to be in the dustbin, as the man alleged they were, and if his story were true, which might have been the case, seeing that he had a character not only for honesty but for being of a "saving nature," there was ground for some explanation.

### THE BRITISH HOSPITALS ASSOCIATION.

We have received a copy of the "Report of a Meeting held at Charing Cross Hospital, London, on July 30, 1915, in reference to the shortage of resident medical officers in the voluntary hospitals." We trust that all hospital men, awake to the importance of the question discussed at that meeting, when, as reported in The Hospital of August 7, Mr. J. C. Buchanan opened a conversation on the subject, will read and preserve the pamphlet. They ought to do so, whether they are members of the British Hospitals Association or not, and an additional reason is perhaps that the pamphlet is practically a verbatim report of the proceedings. For all, and we hope that they are the majority, who are keenly alive to the value which the British Hospitals Association should be to the whole voluntary system at the present time, and who also know, as practical men, how difficult it is, in these busy days, to gather the responsible officers together so as to form a united front and a collective body of opinion, the conversation opened by Mr. Buchanan is to be welcomed, and also side by side with The Hospital's material digest the detailed account of the proceedings which the pamphlet gives. For while a Journal for busy workers actively engaged in the swim of things is read anywhere and everywhere, as the late Dr. Billings knew, the proper place also for Proceedings is the library.

### THE TRAINING OF MEDICAL WOMEN.

An appeal for funds to increase the facilities for the training of medical women has been issued by Earl Curzon of Kedleston, Mr. Asquith, and Mr. Balfour. "The war," they write, "has constituted a turning-point in the position of medical women, and there are new openings and new opportunities for them in many directions. Increasing numbers of women are desirous of entering the profession, and to provide for their adequate educational needs the London (Royal Free Hospital) School of Medicine for Women is now practically doubling its laboratory accommodation. The Council of the School has already received £15,000 of the £30,000 required for the additional buildings and their equipment." There is no doubt that at present there are many openings for qualified medical women. Critics of such schemes of course allege that it is uncertain how far it is wise to take advantage of the exceptional circumstances now existing in order to induce large numbers of women to enter the medical profession. Before the war there was little sign of a large increase in the demand for their services. After the war there is small doubt but that as regards medical affairs matters may revert more or less to the same conditions as were existing before it. From past experience alone it is not easy to see where the new openings and new opportunities for medical women will then be found. Since, however, there is a demand for supplementing the shortage of medical men, and since this naturally attracts many women to the profession, it behoves statesmanlike people so to regulate the new demand as to diminish as far as possible any disappointment which may await many of them when their training is complete. It is also time to see that opportunities are partly made as well as partly found, and the active work undertaken by countless women in medicine and nursing must, to some extent, make their present welcome a more or less permanent one.

### THE ANGLO-RUSSIAN HOSPITAL OR RUSSIAN FLAG DAY FUND?

We noted last week the proposal, supported by Lord Cheylesmore and others, to equip and maintain a hospital with a British staff under the Russian Red Cross. Hot on the heels of this appears a criticism of the plan from Mr. Albert Vickers, chairman of the committee of the Russian Flag Day Fund, who asserts that the objects of Lord Cheylesmore and his friends have been achieved through the sums, amounting to some £20,000, already dispatched to the Russian Red Cross. Mr. Vickers further remarks that early in the war Messrs. Vickers, Ltd., built and equipped at their own expense a hospital for the Russian wounded in Petrograd, and undertook to subscribe towards its maintenance. The crux of his criticism, however, seems to lurk in the suggestion of overlapping, which is implied by Mr. Vickers's statement that "arrangements have now been completed for local celebrations [on behalf of the Russian Flag Day Fund] in practically every city and town in the country, and a large number of centres have already made their collection." Mr. Vickers concludes by an appeal to all who appreciate the help of Russia to subscribe to the fund of which he is chairman, whose address is General Buildings, Aldwych, W.C., and remarks that by so doing the Russian wounded will be helped, without, in effect, the expense of an overlapping organisation. We doubt if the issue can be reduced to such a simple question, a view confirmed by a statement in support of the Anglo-Russian Hospital by Lord Cromer, its president, in the *Times* of the 18th inst.

### THE TRUTH ABOUT OVERLAPPING.

The attitude which is formulated in the question "either my organisation or a waste of your money" is a common and convenient one for the purposes of raising money by subscription, but, in practice, it is as often wrong as right, since overlapping depends as much upon the power of one organisation to do the work required as upon the number attempting to do it, and engaged in a competitive race for subscriptions. In short, the issue really is whether such a hospital as that proposed by Lord Cheylesmore and his supporters is wanted by the Russian Red Cross or not. For nearly all Englishmen this is a matter for legitimate discussion, and therefore, and on the face of it, is the Anglo-Russian Committee, which favours the suggested new hospital, any less likely to be right than the Russian Flag Day Fund? That is the point on which the evidence put forward by the disputants will determine.

### THE BOOM OF THE BLUEBOTTLE.

If the *fons et origo* of disease is being only very gradually demonstrated, the carrier, however, is becoming laid by the heels. A short time ago the oyster enjoyed the public eye, with a view to its appreciators knowing where, for how long, and under what conditions it had slept before being ingested at table. Now the ubiquitous fly is attracting the public attention. Most people now remember that in the South African war mortality from disease was far higher than from wounds, and that enteric fever was for the most part the cause, and the fly to a considerable extent the carrier. The somewhat dormant interest in the merely laborious and painstaking prevention of disease by the direct process of exterminating flies and bluebottles has been aroused by the war. It is consoling to many of us to know that the advent of the mechanically-propelled vehicle, with the corresponding diminution in the number of horses and the amount of their offal, has considerably lessened the number of flies, by depriving them of good and favourable breeding-places. But it is highly necessary to take every precaution to prevent the spread of disease in the large camps that are scattered over Britain and Europe generally. Indeed, the potential danger to the public health from house-flies and blow-flies is still not sufficiently appreciated, and the pamphlet issued by the Zoological Society of London on the subject is very timely. The only risk is the danger of a boom, for, by a noteworthy retribution, any topic "run" by the less scrupulous lay papers is "run to death" with swift inevitableness. The scientific book on the fly, therefore, is to be welcomed.

### THE RESOURCES OF SCIENCE AGAINST THE FLY.

Dr. P. Chalmers Mitchell, secretary to the Zoological Society of London, reminds us in the introduction to his pamphlet on the above subject that his Society early in the present year arranged an exhibition in the Society's gardens to show the dangers to public health due to house-flies and blow-flies, and the methods of dealing with them. Later, the Imperial College of Science joined in the investigation, the Local Government Board made a grant towards the expenses, and the Zoological Society of London and Mr. Otto Beit, F.Z.S., also provided funds. Much experimental work was accomplished, and full scientific reports of these investigations will be published later, but in view of the urgent necessity of immediate action it has been thought well to give immediate advice. The pamphlet called "Practical Advice on the Fly Question" is to be obtained from the Zoological Society of London, Regent's Park, N.W. Single copies, post free, 1½d., 7s. 6d. per 100, 35s. per 500 copies, carriage paid in Great Britain. The pamphlet explains why flies are dangerous, and does not hesitate to remind us that if we do not mind sharing a little filth with the flies, we must also share the seeds of disease which emanate from it. No doubt many educated persons think they know how to catch and kill flies. There are many alternative methods. The fly must be destroyed wholesale, and with the advice given in the pamphlet we can not only choose and experiment with alternative methods and traps, but also learn the best bait, which is casein and brown sugar.

The Military Hospital, Malta.*

### THIS WEEK'S DRUG MARKET.

There is a very active demand for synthetic drugs, the prices of which continue to advance. Acetylsalicylic acid, guaiacol carbonate, resorcin, phenacetin, and salol are among the drugs which are again dearer. Salicylic acid and sodium salicylate are unchanged in price, but any increase in the demand would no doubt result in higher quotations. The prices for bromides are well maintained. Cod-liver oil is unchanged. There is a good demand for mercurials. Quinine has advanced considerably in price. Thymol is extremely scarce, and very high prices are asked for the small quantities available. Acetic acid is dearer. Opium is unchanged. At the recent public auction of drugs in Mincing Lane honey, ipecacuanha, menthol, and sarsaparilla sold at lower rates. Senna fetched still higher prices.

* Note.—We have pleasure in reproducing the above excellent photograph of the military hospital at Malta, which has reached us through the post.

## Figures and Tables

**Figure f1:**